# Message from the New Editor-in-Chief

**DOI:** 10.2188/jea.JE20110142

**Published:** 2012-01-05

**Authors:** Hiroyasu Iso

Dear Colleagues:

I am pleased to announce that the official 2010 impact factor for the Journal of Epidemiology is 2.110. Our journal placed 50th among the 140 journals in the category of Public, Environmental & Occupational Health and top in the Asia-Pacific region. The proportion of self-citation was only 9%, which indicates that ours is a sound and promising journal in epidemiology. The 10 most frequently cited papers in the Journal of Epidemiology in 2008 and 2009 are shown below.

The average time between submission and first response is 27 days. For this achievement, I sincerely thank the editorial committee members and reviewers for their outstanding efforts.

In addition to our regular review articles, we have begun publishing a new series of joint review articles in various research fields, *Epidemiology—Scientific Wisdom and Perspectives from East and West*, which are written by leading epidemiologists from Asian and Western countries.

Our editorial team looks forward to receiving new high-quality submissions from around the world in a broad range of topics in epidemiology. In this way, our journal can continue to advance research in basic and clinical science, public health science, and health policy.

Cordially, Hiroyasu Iso, MD, PhD, MPH Editor-in-Chief Journal of Epidemiology Professor of Public Health Osaka University Graduate School of Medicine

**Figure:**
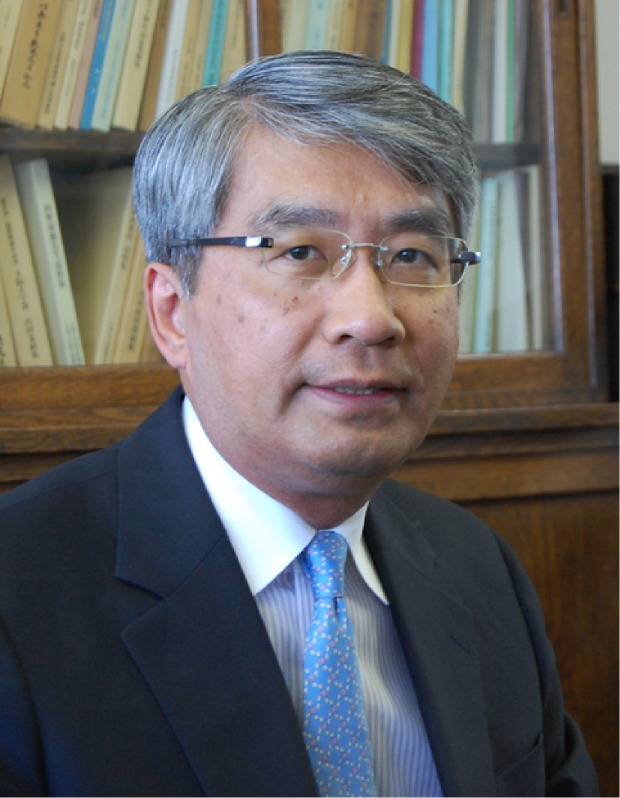


## References

[1] Nakamura Y, Yashiro M, Uehara R, Oki I, Watanabe M, Yanagawa H Epidemiologic features of Kawasaki disease in Japan: Results from the nationwide survey in 2005–2006. J Epidemiol. 2008 Jul;18(4):167–72 10.2188/jea.JE2008001 18635901 PMC4771586

[2] Ishikawa S, Kayaba K, Gotoh T, Nago N, Nakamura Y, Tsutsumi A, Kajii E Incidence of total stroke, stroke subtypes, and myocardial infarction in the Japanese population: The JMS cohort study. J Epidemiol. 2008 Jul;18(4):144–50 10.2188/jea.JE2007438 18603825 PMC4771583

[3] Lin Y, Kikuchi S, Tamakoshi K, Wakai K, Kondo T, Niwa Y, Yatsuya H, Nishio K, Suzuki S, Tokudome S, Yamamoto A, Toyoshima H, Mori M, Tamakoshi A Active smoking, passive smoking, and breast cancer risk: Findings from the Japan collaborative cohort study for evaluation of cancer risk. J Epidemiol. 2008 Mar;18(2):77–83 10.2188/jea.18.77 18403857 PMC4771580

[4] Katanoda K, Marugame T, Saika K, Satoh H, Tajima K, Suzuki T, Tamakoshi A, Tsugane S, Sobue T Population Attributable Fraction of Mortality Associated with Tobacco Smoking in Japan: A Pooled Analysis of Three Large-scale Cohort Studies. J Epidemiol. 2008 Nov;18(6):251–64 10.2188/jea.JE2007429 19075498 PMC4771610

[5] Sadakane A, Tsutsumi A, Gotoh T, Ishikawa S, Ojima T, Kario K, Nakamura Y, Kayaba K Dietary patterns and levels of blood pressure and serum lipids in a Japanese population. J Epidemiol. 2008 Mar;18(2):58–67 10.2188/jea.18.58 18403855 PMC4771578

[6] Narisawa S, Nakamura K, Kato K, Yamada K, Sasaki J, Yamamoto M Appropriate waist circumference cutoff values for persons with multiple cardiovascular risk factors in Japan: a large cross-sectional study. J Epidemiol. 2008 Jan;18(1):37–42 10.2188/jea.18.37 18305365 PMC4771601

[7] Suzuki K, Tanaka T, Kondo N, Minai J, Sato M, Yamagata Z Is maternal smoking during early pregnancy a risk factor for all low birth weight infants? J Epidemiol. 2008 May;18(3):89–96 10.2188/jea.JE2007415 18469489 PMC4771603

[8] Zhou Z, Hu D An epidemiological study on the prevalence of atrial fibrillation in the Chinese population of mainland China. J Epidemiol. 2008 Sep;18(5):209–16 10.2188/jea.JE2008021 18776706 PMC4771592

[9] Ishiguro C, Fujita T, Omori T, Fujii Y, Mayama T, Sato T Assessing the effects of non-steroidal anti-inflammatory drugs on anti hypertensive drug therapy using post-marketing surveillance database. J Epidemiol. 2008 May;18(3):119–24 10.2188/jea.JE2007413 18469490 PMC4771606

[9] Wakai K A Review of Food Frequency Questionnaires Developed and Validated in Japan. J Epidemiol. 2009 Jan;19(1):1–11 10.2188/jea.JE20081007 19164867 PMC3924089

[10] Nishio K, Goto Y, Kondo T, Ito S, Ishida Y, Kawai S, Naito M, Wakai K, Hamajima N Serum folate and methylenetetrahydrofolate reductase (MTHFR) C677T polymorphism adjusted for folate intake. J Epidemiol. 2008 May;18(3):125–31 10.2188/jea.JE2007417 18480590 PMC4771607

[10] Ozasa K, Katanoda K, Tamakoshi A, Sato H, Tajima K, Suzuki T, Tsugane S, Sobue T Reduced life expectancy due to smoking in large-scale cohort studies in Japan. J Epidemiol. 2008 May;18(3):111–8 10.2188/jea.JE2007416 18480591 PMC4771605

